# Validity and reliability of the Dutch version of the Copenhagen Hip And Groin Outcome Score (HAGOS-NL) in patients with hip pathology

**DOI:** 10.1371/journal.pone.0186064

**Published:** 2017-10-11

**Authors:** Hilde Giezen, Martin Stevens, Inge van den Akker-Scheek, Inge H. F. Reininga

**Affiliations:** 1 Department of Orthopedics, University of Groningen, University Medical Center Groningen, Groningen, The Netherlands; 2 Department of Trauma Surgery, University of Groningen, University Medical Center Groningen, Groningen, The Netherlands; 3 Department of Orthopedics, Martini Hospital Groningen, Groningen, The Netherlands; University of Michigan, UNITED STATES

## Abstract

**Background and objectives:**

The Copenhagen Hip And Groin Outcome Score (HAGOS) was developed to assess disease-specific consequences in young to middle-aged, physically active hip and/or groin patients. The study aimed to determine validity and reliability of the Dutch version of the HAGOS (HAGOS-NL) for middle-aged patients with hip complaints.

**Design and methods:**

To assess validity, 117 participants completed five questionnaires: HAGOS-NL, international Hip Outcome Tool (iHOT-12NL), Hip disability and Osteoarthritis Outcome Score (HOOS), RAND-36 Health Survey and Tegner activity scale. Structural validity was determined by conducting confirmatory factor analysis. Construct validity was analyzed by formulating predefined hypotheses regarding relationships between the HAGOS-NL and subscales of the iHOT-12NL, HOOS, RAND-36 and Tegner activity scale. The HAGOS-NL was filled out again by 67 patients to explore test-retest reliability. Reliability was assessed in terms of Cronbach’s alpha, Intraclass Correlation Coefficient (ICC), Standard Error of Measurement (SEM) and Minimal Detectable Change (MDC). The Bland and Altman method was used to explore absolute agreement.

**Results:**

Factor analysis confirmed that the HAGOS-NL consists of six subscales. All hypotheses were confirmed, indicating good construct validity. Internal consistency was good, with Cronbach’s alpha values ranging from 0.89 to 0.98. Test-retest reliability was considered good, with ICC values of 0.80 and higher. The SEM ranged from 6.6 to 12.3, and MDC at individual level from 18.3 to 34.1 and at group level from 2.3 to 4.4. Bland and Altman analyses showed no bias.

**Conclusion:**

The HAGOS-NL is a reliable and valid instrument for measuring pain, physical functioning and quality of life in middle-aged patients with hip complaints.

## Introduction

Musculoskeletal pain is particularly common among the elderly population [1]. A substantial part of these musculoskeletal complaints is hip and/or groin pain. Approximately 22% of the Dutch elderly population (ages 65+) experiences hip complaints, with a point prevalence of 17% [2]. However, also people under the age of 65 are affected, with an incidence of approximately 11% [3].

Hip complaints worsen physical functioning and health-related quality of life [4]. To get an impression of these disease-specific consequences, including physical functioning and health-related quality of life, a patient-reported outcome measure (PROM) is often used. Such questionnaires are already available for elderly patients with hip complaints. It is questionable if these questionnaires are also suitable for middle-aged hip patients, as physical activity performance goals differ from elderly hip patients [5–7].

A disease-specific questionnaire that focuses more on the physical activity performance goals of younger hip patients was developed by Mohtadi et al. (2012): the international Hip Outcome Tool 33 (iHOT-33) [8]. The short version of the iHOT-33 is validated and translated into Dutch: the iHOT-12NL [9]. The iHOT-12NL is a reliable and valid instrument for measuring physical functioning and health-related quality of life in younger patients with hip pathology [9].

Another questionnaire developed for assessment of disease-specific consequences in younger to middle-aged patients with hip and/or groin pain is the Copenhagen Hip and Groin Outcome Score (HAGOS). The HAGOS consists of six subscales: Symptoms, Pain, Physical function in daily living (ADL), Function in sport and recreation, Participation in physical activities (PA), and Hip-related quality of life (QOL) [10]. Althoug the HAGOS was originally developed for young to middle-aged patients with hip and/or groin pain, it is questionable whether the HAGOS can also be used in a general population of middle-aged patients with hip complaints, like for example patients with osteoarthritis or hip dysplasia.

The HAGOS is currently available in English, Danish and Swedish [10,11]. Recently, Brans et al. (2016) translated the HAGOS into Dutch (HAGOS-NL) by forward and backwards translation and testing of cross-cultural validity. Their study showed that the HAGOS-NL has sufficient validity and reliability [12]. However, as their study population only consisted of middle-aged patients who had undergone a groin hernia repair, it needs to be investigated whether the HAGOS-NL is also valid en reliable in middle-aged patients with hip complaints. Therefore, the aim of this study was to determine validity and reliability of the HAGOS-NL for that population.

## Methods

### Study design and population

In this study the validity and reliability of the HAGOS-NL in patients with hip complaints was investigated. The study was conducted between January and July 2014 at the orthopedic departments of University Medical Center Groningen (UMCG) and Martini Hospital Groningen. The study was reviewed and approved by the Medical Ethical Committee (METC) of UMCG (January 21, 2014; no. 2013.514). (Parts) of the study methods have been described in detail previously [9,13]. Eligible patients were between 18 and 60 years old and were treated for various pathologies of the hip:

- Hip dysplasia
- Osteoarthritis of the hip treated with conservative treatment, hip arthroscopy or total or partial hip replacement (maximum one and a half year postoperative), including patients who were on the waiting list
- Avascular necrosis of the femoral head treated with total or partial hip replacement, including patients who were on the waiting list
- Traumatic hip fracture treated with total or partial hip replacement

All included participants completed an informed consent form prior to participation.

### Measurement procedure

To determine validity of the HAGOS-NL, participants were asked to complete five questionnaires: HAGOS-NL [12], iHOT-12NL [9], HOOS [14,15], RAND-36 [16,17] and the Tegner activity scale [18]. All questionnaires were sent to the participants by mail, together with an information letter explaining the procedure and purpose of the study.

To explore test-retest reliability, participants were asked to complete the HAGOS-NL again after two weeks. This 2-week interval was considered adequate to prevent recall bias and to ensure that clinical change had not occurred [19]. To detect a possible clinical change in the interval between the two administrations, patients answered a Global Rating of Change (GRC) question: “Has there been a change in your hip complaints, compared to two weeks ago?” The GRC question was answered with a 6-point Likert scale, ranging from “much improved” to “worse than ever”. Patients who reported being much improved or much deteriorated were excluded from reliability analyses. Patients who underwent total hip replacement had to be at least six months postoperative at the time the HAGOS-NL was first administered. At six months following total hip replacement patients were likely to be in a stable state of recovery so no change in their physical functioning was expected between the first and second administration of the HAGOS-NL.

### Questionnaires

#### HAGOS-NL

The HAGOS is a disease-specific questionnaire for the assessment of symptoms, activity limitations, participation restrictions and quality of life in young to middle-aged, physically active patients with longstanding hip and/or groin pain [10]. A Dutch version of the HAGOS (HAGOS-NL) is available [12]. The HAGOS-NL consists of six subscales: Symptoms in hip and/or groin; Pain in hip and/or groin; Function in daily living (ADL); Function in sport and recreation; Participation in physical activities; and Hip and/or groin-related quality of life. The questions can be answered using a 5-point Likert scale. For each subscale a sum score is calculated and converted into a 100-point scale. Higher scores reflect less pain and disability [10].

#### iHOT-12NL

The iHOT-12 is a questionnaire that measures physical functioning and health-related quality of life in younger, physically active patients with hip pathology. A valid and reliable Dutch version (iHOT-12NL) is available [9]. The iHOT-12NL consists of 12 questions that are answered by placing a mark on a 100-mm scale, where scores range from 0 to 100, with 100 representing the best possible quality-of-life score. The total score is calculated as a mean of all responded questions [20].

#### HOOS

The HOOS is a questionnaire that assesses the patients’ opinion about their hip and associated problems. A valid and reliable Dutch version is available [14]. The HOOS consists of five subscales: 1) Pain; 2) Symptoms; 3) Function in daily living (ADL); 4) Function in sport and recreation; and 5) Hip-related quality of life. Questions are answered using a 5-point Likert scale. For each subscale, a sum score is calculated which is then converted to a normalized score ranging from 0 to 100, where a lower score reflects more symptoms [14,15].

#### RAND-36

The RAND-36 is a generic questionnaire that assesses health status and health-related quality of life. A valid and reliable Dutch version is available [17]. The RAND-36 includes 36 questions that assess eight health concepts: 1) limitations in physical activities because of health problems (PF); 2) limitations in social activities because of physical or emotional problems (SF); 3) limitations in usual role activities because of physical health problems (RP); 4) bodily pain (BP); 5) general mental health (MH); 6) limitations in role activities because of emotional problems (RE); 7) vitality (VT); and 8) general health perceptions (GH). For each subscale a sum score is calculated and converted into a 100-point scale. A higher score indicates better health [16].

#### Tegner activity scale

The Tegner activity scale is a grading scale where the level of activities in daily living, recreation and competitive sports can be graded numerically. The patient chooses one of the 11 levels that best matches his/her level of daily activities. The corresponding level forms the final score, where a higher score indicates a higher level of daily activities. Patients with Tegner level 4 or higher are defined as physically active [18].

### Validity

Structural and construct validity of the HAGOS-NL were assessed. Structural validity is the degree to which the score of an instrument adequately reflects the dimensionality of the construct being measured. Construct validity is the extent to which scores on a particular instrument relate to other measures, in accordance with predefined hypotheses concerning measured constructs [19,21]. According to the COSMIN guidelines [21], 14 predefined hypotheses were formulated about the magnitude of the relationship between the HAGOS-NL and subscales of the iHOT-12NL, HOOS, RAND-36, and Tegner activity scale (Table 1).

**Table 1:** Predefined and confirmed hypotheses.

1	Correlation ≥0.60 between the HAGOS subscales *Symptoms*, *Pain*, *ADL*, *Sport and recreation*, and *Quality of life* and the iHOT-12NL.
2	Correlation ≥0.40 between the HAGOS subscale *Participation in physical activities* and the iHOT-12NL.
3	Correlation ≥0.90 between the HAGOS subscales *Symptoms* and HOOS subscale *Symptoms*.
4	Correlation ≥0.90 between the HAGOS subscale *Pain* and HOOS subscale *Pain*.
5	Correlation ≥0.80 between the HAGOS subscale *ADL* and HOOS subscale *ADL*.
6	Correlation ≥0.80 between the HAGOS subscale *Function in sport and recreation* and HOOS subscale *Function in sport and recreation*.
7	Correlation ≥0.90 between the HAGOS subscale *Quality of life* and HOOS subscale *Quality of life*.
8	Correlation ≥0.50 between HAGOS subscale *Symptoms* and RAND-36 subscale *Physical functioning*, *Role-physical limitations* and *Bodily pain*.
9	Correlation ≥0.60 between HAGOS subscale *Pain* and RAND-36 subscale *Physical functioning* and *Bodily pain*
10	Correlation ≥0.60 between HAGOS subscale *ADL* and RAND-36 subscale *Physical functioning*, *Role-physical limitations* and *Bodily pain*.
11	Correlation ≥0.5 between HAGOS subscale *Function in sport and recreation* and RAND-36 subscale *Physical functioning*, *Role-physical limitations* and *Bodily pain*.
12	Correlation ≥0.5 between HAGOS subscale *Quality of life* and RAND-36 subscale *Physical functioning*, *Role-physical limitations* and *Bodily pain*.
13	Correlation ≥0.6 between HAGOS subscale *Participation in physical activity* and RAND-36 subscale *Role-physical limitations*.
14	Patients with Tegner level ≥4 score significantly higher on HAGOS-NL subscales *Function in sport and recreation* and *Participation in physical activities* than patients with Tegner level <4.

For the relationship between the Swedish versions of the HAGOS and iHOT-12, Thomee et al. (2014) expected correlations of 0.5 and higher, given that the HAGOS and iHOT-12 are developed for similar patient groups and essentially measure the same constructs [11]. Based on the correlations found in the study of Thomee et al. (2014) and Brands et al. (2016), the current study expected correlations of 0.6 and higher for all subscales of the HAGOS-NL with the iHOT-12NL [11,12]. As Brans et al. (2016) showed a moderate correlation for the subscale *Participation in physical activities*, *a* smaller correlation of 0.4 and higher was expected for this subscale [12].

Since the HAGOS was developed to measure physical functioning rather than social and/or emotional aspects, relatively smaller correlations were expected between the HAGOS subscales and the RAND-36 subscales that tend to measure social and/or emotional aspects. For the subscales of the HAGOS that measure physical functioning, moderate-to-strong correlations were expected with subscales of the RAND-36 that are also related to physical functioning. These expectations are in line with those stated in the study of Brans et al. (2016) [12]. Moreover, as the HAGOS is based on and designed similarly to the HOOS, strong correlations were expected between the subscales of these questionnaires. Since the HAGOS was originally designed for physically active patients, these patients (Tegner level ≥4) were expected to score significantly higher on the HAGOS-NL subscales *Function in sport and recreation* and *Participation in physical activities* than patients with Tegner level <4. Construct validity can be considered good when at least 75% of the hypotheses are confirmed [19].

### Reliability

Following the COSMIN guidelines [21], reliability was assessed in terms of internal consistency, test-retest reliability and measurement error. Internal consistency refers to the extent to which subscales of a questionnaire are related; test-retest reliability concerns the extent to which patients’ scores are the same for repeated measurements; and measurement error is a measure of systematic error of a patient’s score that is not caused by actual changes in the measured construct [21]. The Bland and Altman method was used to explore absolute agreement, which reflects the amount of agreement in repeated measurements [22].

### Floor and ceiling effects

To assess the depth of the health measures, floor and ceiling effects were analyzed by calculating the prevalence of the lowest and highest possible scores [23].

### Statistical analysis

A sample size with a minimum of 100 subjects is required for studies on measurement properties of questionnaires with factor analyses [21], and a sample size of 50 is considered adequate for determining test-retest reliability [24]. Hence we planned a sample size of at least 100 participants to assess construct validity and a sample size of at least 50 to establish test-retest reliability of the HAGOS-NL. All statistical analyses were performed using SPSS version 22.0 for Windows (SPSS, Armonk, NY, USA). A P-value <0.05 was considered to indicate statistical significance.

To assess structural validity, principal component confirmatory factor analyses with varimax rotation were conducted on all individual subscales. To analyze construct validity, Spearman’s correlation coefficients between the subscales of the HAGOS-NL and (subscales of) the iHOT-12NL, HOOS, RAND-36 and Tegner activity scale were calculated. Spearman’s correlations were interpreted according to Domholdt: 0.00 to 0.25 very weak; 0.26 to 0.49 weak; 0.50 to 0.69 moderate; 0.70 to 0.89 strong; and 0.90 to 1.00 very strong [25].

To evaluate whether physically active participants (Tegner level ≥4) have a higher score on the HAGOS-NL subscales *Function in sport and recreation* and *Participation in physical activities* than participants who are not physically active (Tegner level <4), a Mann-Whitney U-test was conducted.

Internal consistency was assessed by calculating Cronbach’s alpha for each subscale of the HAGOS-NL. Values between 0.70 and 0.95 are considered to indicate good internal consistency [19]. To analyze test-retest reliability, Intraclass Correlation Coefficients (ICCs) with 95% Confidence Interval (CI) were calculated for each subscale using a two-way random effects model, type absolute agreement. According to Terwee et al. (2007), ICC values of 0.70 or higher indicate high test-retest reliability. Measurement error was analyzed by calculating the Standard Error of Measurement (SEM) and the Minimal Detectable Change (MDC) [19]. SEM was calculated by multiplying the pooled standard deviation by √(1-r), where r is the ICC.[26] From the SEM, the MDC at the individual level (MDC_ind_) was calculated using the formula 1.96 × SEM × √2 and at the group level by dividing MDC_grp_ by √n [19]. To assess absolute agreement between the first and second administration of the HAGOS-NL, the Bland and Altman method was used [22]. The mean difference between the first and second administration of all HAGOS-NL subscales with 95% CI were calculated. Zero lying within the 95% CI of the mean difference was considered a criterion for absolute agreement. In addition, the 95% limits of agreement (LOA) were calculated with the formula mean difference ± 1.96 × SD_diff_, where SD_diff_ is the standard deviation of the mean difference between the first and second administration of the HAGOS-NL.

The prevalence of floor and ceiling effects was evaluated. Questionnaires met standards when the prevalence of floor or ceiling effects was smaller than 15% [23].

## Results

### Descriptive statistics

**Fig 1:**
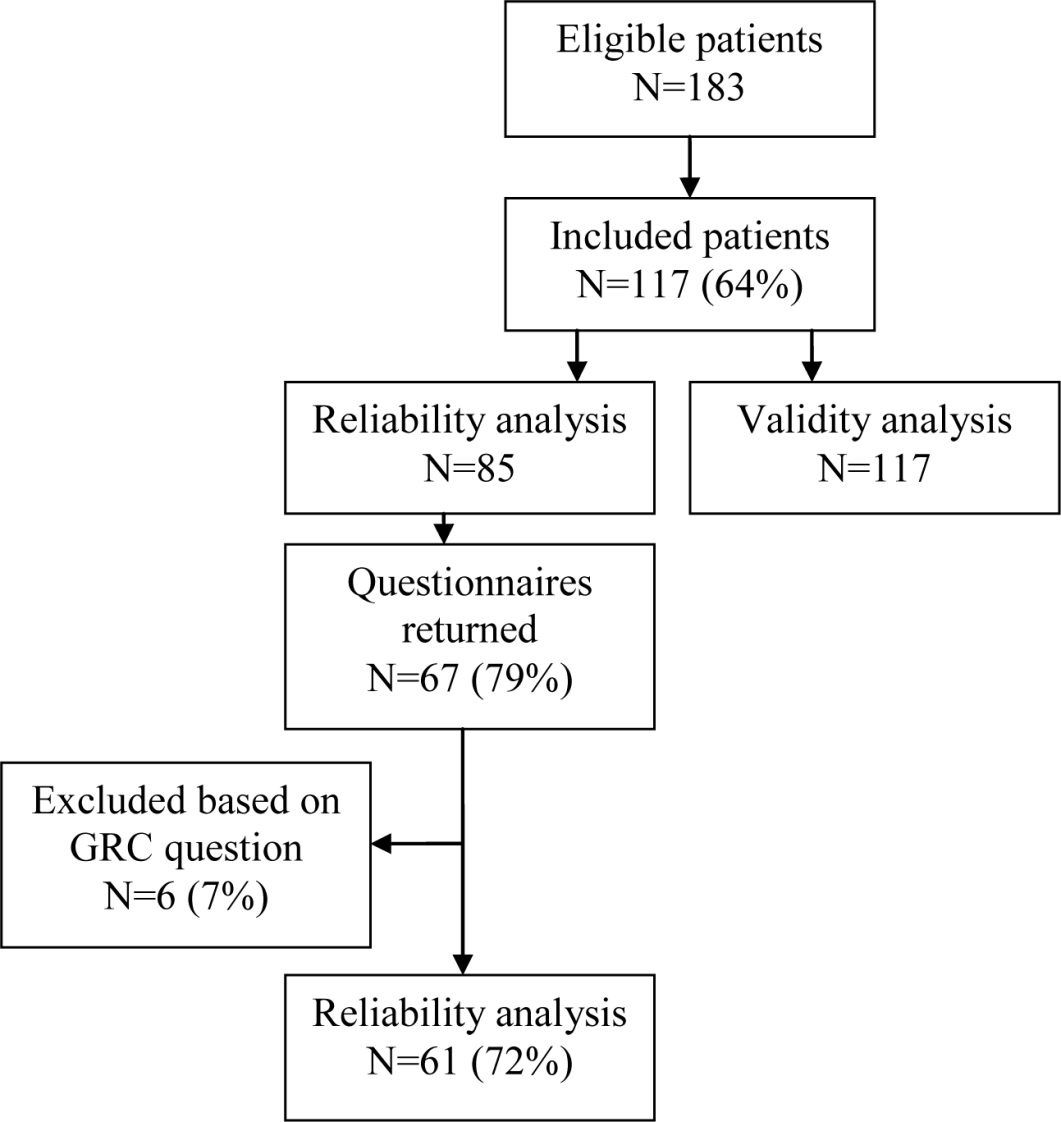
Flow diagram of inclusion of participants. Abbreviation: GRC, global rating of change.

For this study, 183 eligible patients were invited to participate (Fig 1); 117 participants (64%) returned their completed questionnaires. Characteristics of these participants are shown in Table 2.

**Table 2:** Patient characteristics (N = 117).

Characteristics		N (%)
Age (years)* (N = 117)		51 ± 9 (18–60)
Gender (N = 117)		
	Male	40 (34)
	Female	77 (66)
Marital status (N = 117)		
	Single	23 (20)
	With partner	46 (39)
	With partner and children	41 (35)
	With children	5 (4)
	With parents	2 (2)
Educational level (N = 116)		
	Elementary school	30 (26)
	High school	51 (44)
	Higher education	34 (29)
	Other	1 (1)
Affected hip (N = 116)		
	Right	46 (40)
	Left	58 (50)
	Both	12 (10)
Tegner level (N = 117)		
	Tegner level 0–3	81 (69)
	Tegner level 4	26 (22)
	Tegner level 5	7 (6)
	Tegner level >5	3 (3)
Treatment/indication		
	Osteoarthritis conservative treatment	7 (6)
	Hip arthroscopy	11 (9)
	Avascular necrosis	3 (3)
	Hip dysplasia	4 (3)
	Hip fracture	3 (3)
	THA	83 (71)
	On waiting list for THA	6 (5)
Comorbidities (N = 117)		
	Migraine or regular severe headaches	14 (12)
	Hypertension	22 (19)
	Asthma, chronic bronchitis, pulmonary emphysema or COPD	20 (17)
	Severe or persistent indigestion >3 months	4 (3)
	Degeneration of the hip or knee joints	80 (68)
	Chronic inflammatory arthritis	17 (15)
	Severe or persistent back disorders (including hernia)	17 (15)
	Diabetes mellitus	10 (9)
	Heart attack	1 (1)
	Other serious cardiac condition	3 (3)

*Age is reported as mean ± standard deviation (range).

To evaluate reliability of the HAGOS-NL, 85 participants were invited to fill in the questionnaire for a second time after an interval of two weeks. Thirty-two patients with a total hip replacement were not invited as they were not yet 6 months postoperative at the time of the second administration. Eventually, 67 questionnaires (79%) were returned. Based on the GRC question, six participants were excluded from reliability analysis, as one reported that his/her hip complaints were much deteriorated and the other five participants reported that their hip complaints were much improved. Reliability analysis was therefore conducted with 61 patients (72%).

### Validity

Factor analysis for each individual subscale confirmed the structure of six subscales of the HAGOS. Explained variances ranged from 65 to 90% (Table 3).

**Table 3:** Structural validity measures of the HAGOS-NL.

HAGOS-NL subscale	Eigenvalue	Variance explained
Symptoms	4.8	69.0%
Pain	7.0	70.6%
ADL	4.0	79.4%
Sport	6.2	77.7%
PA	1.7	86.3%
QOL	3.8	76.3%

Abbreviations: Symptoms, symptoms in hip and/or groin; Pain, pain in hip and/or groin; ADL, function in daily living; Sport, function in sport and recreation; PA, participation in physical activities; QOL, hip and/or groin-related quality of life

Spearman correlations between the HAGOS-NL subscales and the total score of the iHOT-12NL are shown in Table 4. Moderate-to-high correlations were found, except for the HAGOS-NL subscale *Participation in physical activities*, which correlated weakly. Table 4 also presents the Spearman correlations between the HAGOS-NL and subscales of the HOOS and RAND-36. Except for the HAGOS-NL subscale *Participation in physical activities*, high correlations were observed with HOOS subscales that measure similar constructs. Similarly, higher correlations were found between the HAGOS-NL and RAND-36 subscales PF, PR, and BP than between the HAGOS-NL subscales and RAND-36 subscales MH, VT, RE, SF, and GH.

**Table 4:** Spearman’s correlation coefficients between HAGOS-NL subscales and iHOT-12NL, HOOS subscales and RAND-36 subscales.

	HAGOS-NL					
	Symptoms	Pain	ADL	Sport	PA	QOL
iHOT-12NL	0.87	0.87	0.86	0.84	0.65	0.83
HOOS Symptoms	0.93	0.83	0.81	0.77	0.52	0.80
HOOS Pain	0.90	0.95	0.90	0.85	0.67	0.87
HOOS ADL	0.87	0.91	0.95	0.86	0.65	0.85
HOOS Sport	0.78	0.80	0.82	0.95	0.65	0.80
HOOS QOL	0.81	0.83	0.80	0.81	0.68	0.92
RAND-36 PF	0.73	0.76	0.81	0.79	0.64	0.76
RAND-36 SF	0.58	0.62	0.59	0.58	0.57	0.70
RAND-36 RP	0.62	0.66	0.69	0.56	0.57	0.72
RAND-36 RE	0.26	0.29	0.33	0.27	0.33	0.41
RAND-36 MH	0.38	0.34	0.31	0.28	0.24	0.40
RAND-36 VT	0.54	0.54	0.56	0.56	0.45	0.61
RAND-36 BP	0.74	0.76	0.76	0.71	0.59	0.75
RAND-36 GH	0.38	0.38	0.43	0.35	0.36	0.42

Abbreviations: Symptoms, symptoms in hip and/or groin; Pain, pain in hip and/or groin; ADL, function in daily living; Sport, function in sport and recreation; PA, participation in physical activities; QOL, hip and/or groin-related quality of life; PF, Physical Functioning; SF, Social Functioning; RP, Role-Physical; RE, Role-Emotional; MH, Mental Health; VT, Vitality; BP, Bodily Pain; GH, General Health.

Comparing scores between patients with low and high activity levels revealed that patients with high activity levels (Tegner activity level ≥4) scored significantly higher than patients with lower activity levels (Tegner activity level <4) on the subscales *Sport and recreation* and *Participation in physical activities* of the HAGOS-NL. All predefined hypotheses on the magnitude of relationships between the HAGOS-NL and the iHOT-12NL, HOOS, RAND-36, and Tegner activity scale were confirmed.

### Reliability

**Fig 2:**
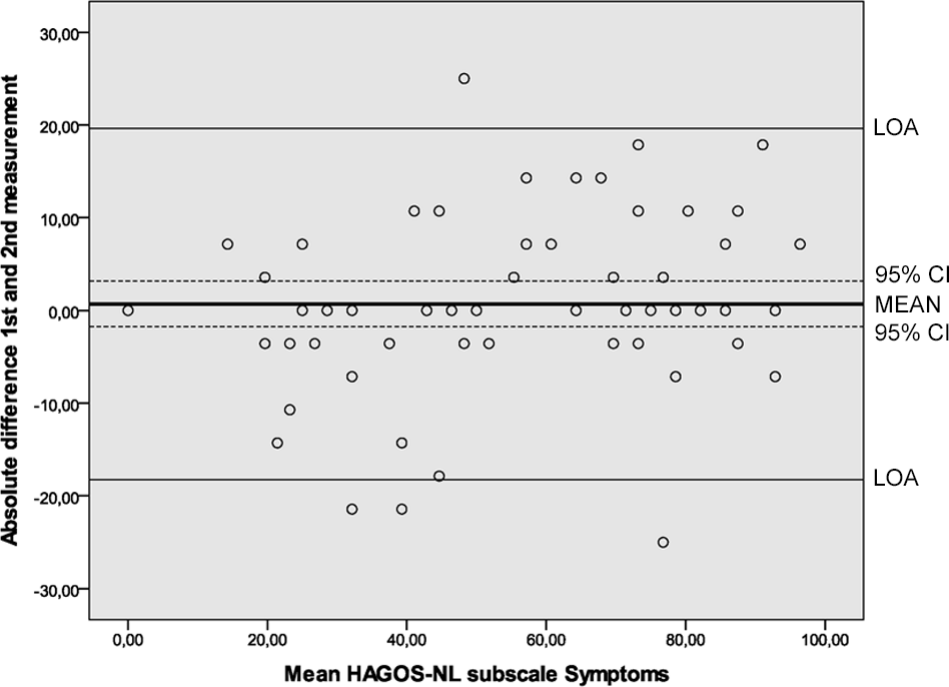
Bland and Altman graph of HAGOS subscale *Symptoms*. Abbreviations: HAGOS, Copenhagen Hip And Groin Outcome Score; LOA, limits of agreement; 95% CI, 95% confidence interval.

**Fig 3:**
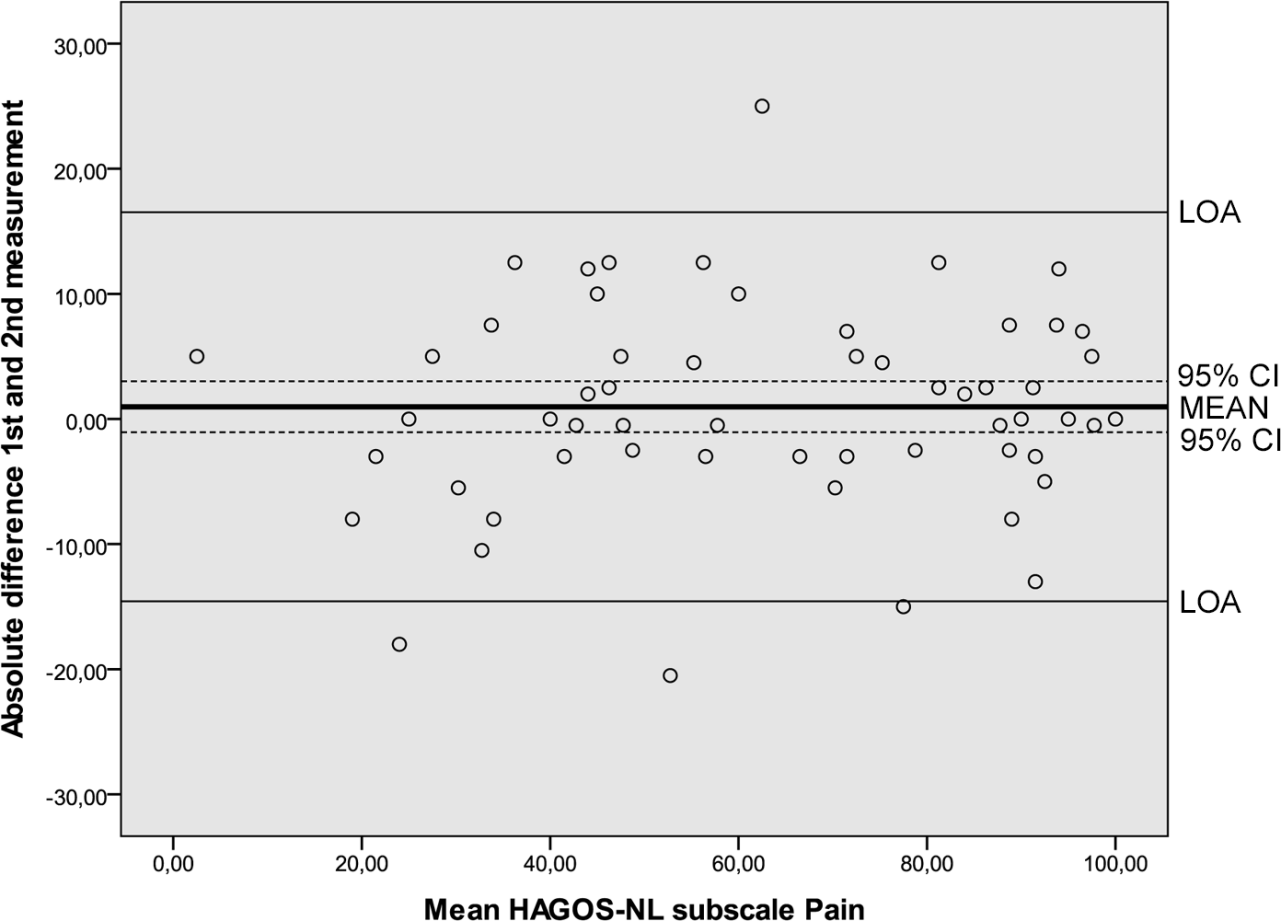
Bland and Altman graph of HAGOS subscale *Pain*. Abbreviations: HAGOS, Copenhagen Hip And Groin Outcome Score; LOA, limits of agreement; 95% CI, 95% confidence interval.

**Fig 4:**
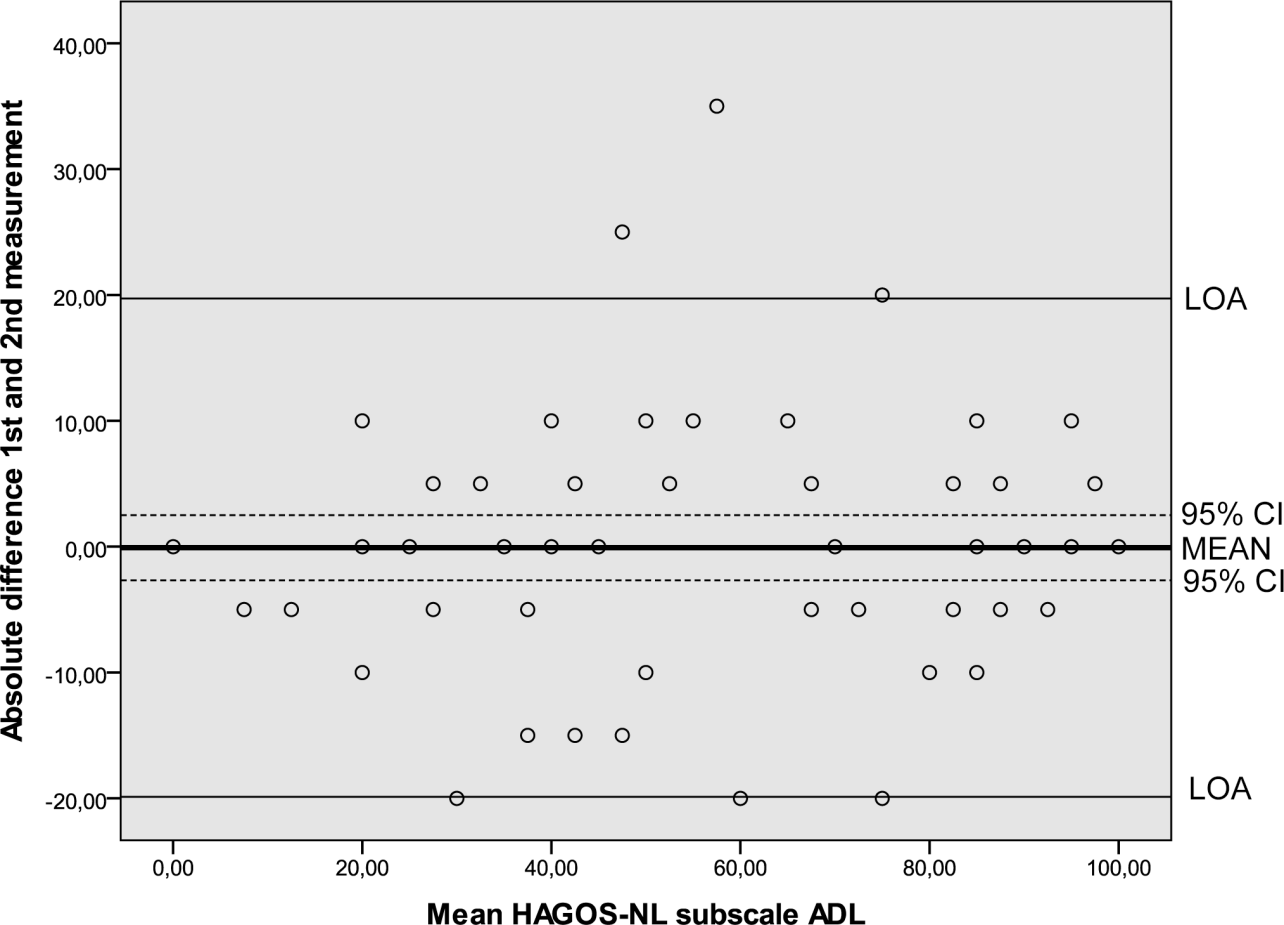
Bland and Altman graph of HAGOS subscale *ADL*. Abbreviations: ADL, Activities of daily living; HAGOS, Copenhagen Hip And Groin Outcome Score; LOA, limits of agreement; 95% CI, 95% confidence interval.

**Fig 5:**
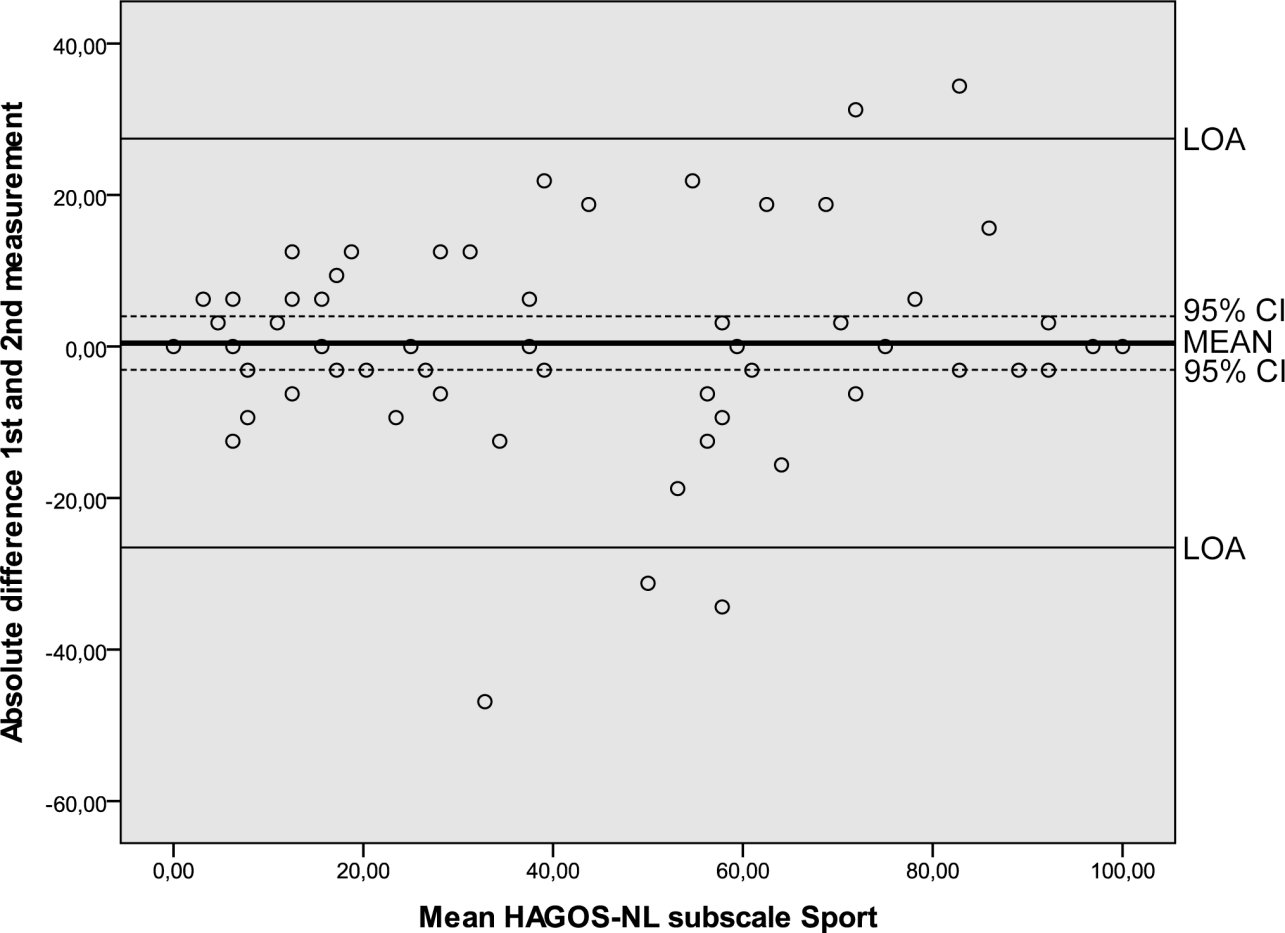
Bland and Altman graph of HAGOS subscale *Sport*. Abbreviations: HAGOS, Copenhagen Hip And Groin Outcome Score; LOA, limits of agreement; 95% CI, 95% confidence interval.

**Fig 6:**
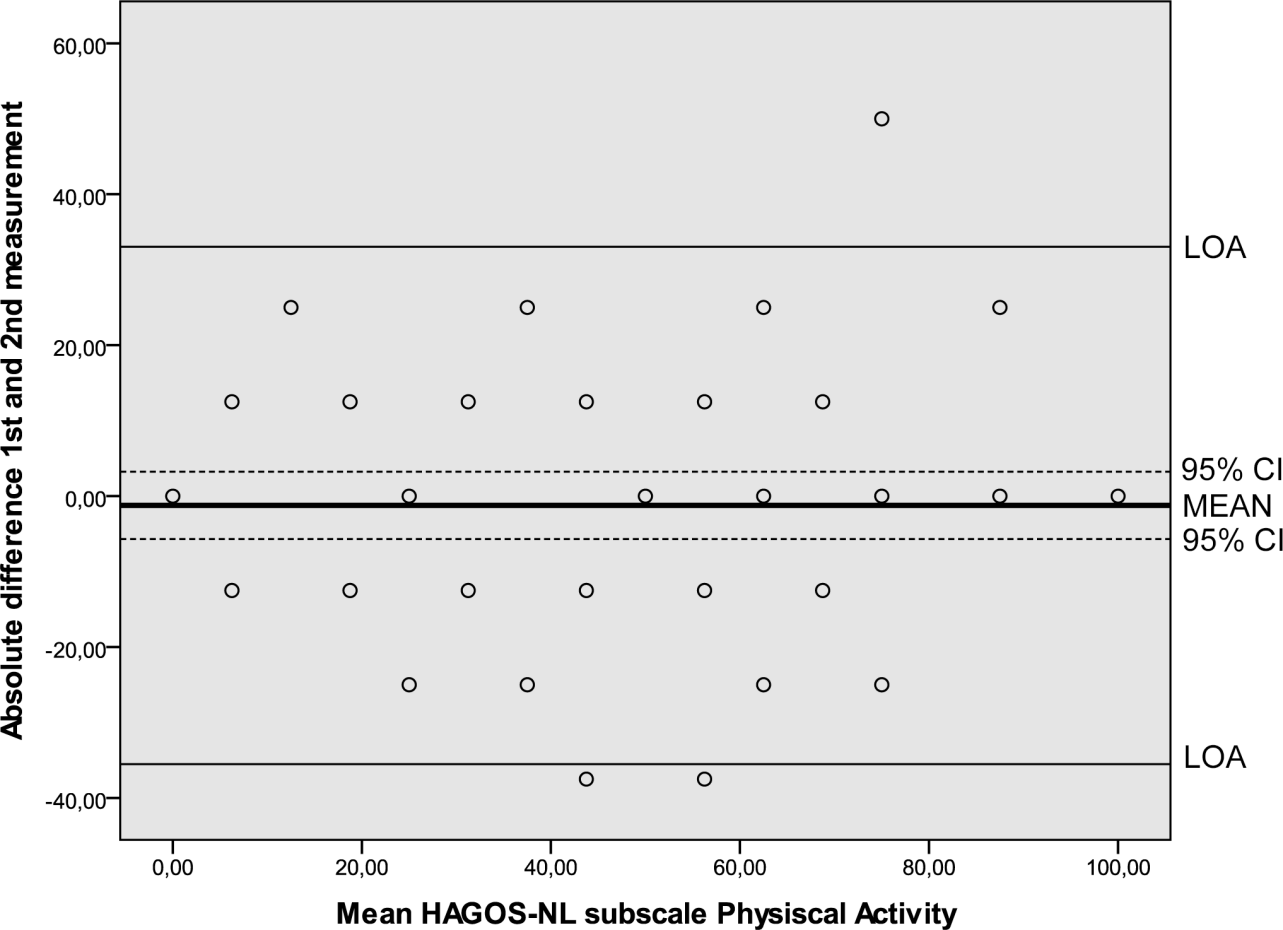
Bland and Altman graph of HAGOS subscale *Functioning in physical activities*. Abbreviations: HAGOS, Copenhagen Hip And Groin Outcome Score; LOA, limits of agreement; 95% CI, 95% confidence interval.

**Fig 7:**
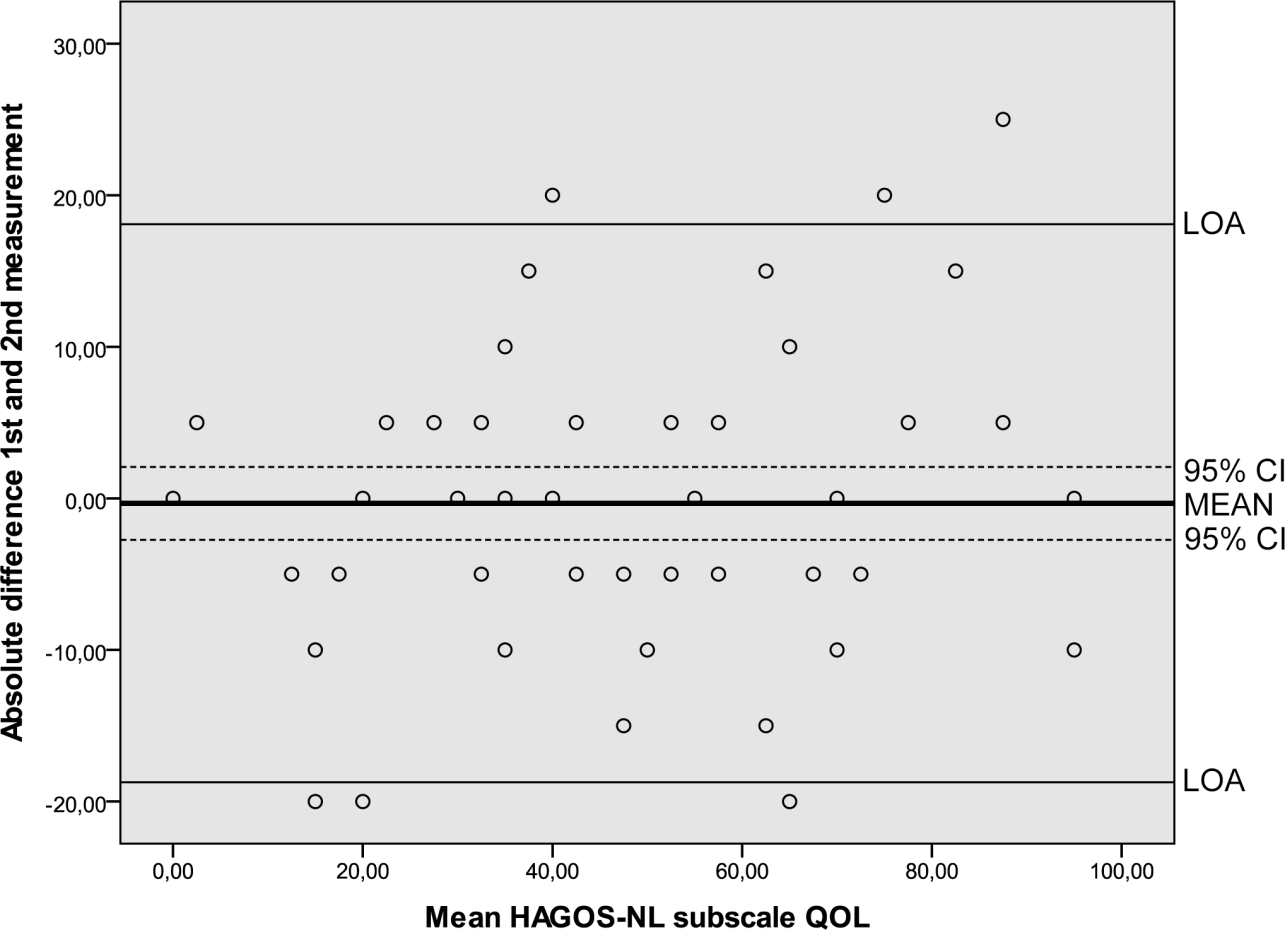
Bland and Altman graph of HAGOS subscale *Quality of Life*. Abbreviations: HAGOS, Copenhagen Hip And Groin Outcome Score; LOA, limits of agreement; QOL, Quality of life; 95% CI, 95% confidence interval.

Results of the reliability analyses are presented in Table 5. Cronbach’s alpha values ranging from 0.89 to 0.97 indicated good internal consistency. ICC’s ranging from 0.80 to 0.95 were found. SEM values ranged from 6.6 to 12.3. MDC values at the individual level ranged from 18.3 to 34.1 and at the group level from 2.3 to 4.4. The Bland and Altman analysis showed zero lying within the 95% CI of the mean difference between the first and second administration of the HAGOS-NL, ruling out systematic bias (Table 5, Figs 2–7).

**Table 5:** Reliability measures of the HAGOS-NL for each subscale (N = 61).

	1^st^ adm. Mean (SD)	2^nd^ adm. Mean (SD)	Mean diff.	(95% CI)	Cronbach alpha	ICC (95%CI)	SEM	MDC ind	MDC grp
Symptoms	56.2 (26.0)	55.4 (23.5)	-0.7	-1.8–3.2	0.96	0.92 (0.88–0.95)	6.8	18.9	2.4
Pain	63.0 (26.0)	62.0 (25.4)	1.0	-1.1–3.0	0.98	0.95 (0.92–0.97)	9.1	25.2	3.2
ADL	58.1 (28.3)	58.2 (27.4)	-0.1	-2.7–2.5	0.97	0.94 (0.90–0.96)	7.1	19.7	2.5
Sport	42.4 (30.9)	42.0 (29.6)	0.5	-3.1–4.0	0.95	0.90 (0.84–0.94)	9.7	26.8	3.4
PA	39.8 (27.8)	41.0 (27.5)	-1.2	-5.7– .2	0.89	0.80 (0.69–0.88)	12.3	34.1	4.4
QOL	46.3 (25.1)	46.6 (23.2)	-0.3	-2.7–2.1	0.96	0.93 (0.88–0.96)	6.6	18.3	2.3

Abbreviations: ICC, Intraclass Correlation Coefficient; 95% CI, 95% Confidence Interval; SEM, standard error of measurement; MDCgrp, smallest detectable change at the group level; MDCind, smallest detectable change at the individual level; Symptoms, symptoms in hip and/or groin; Pain, pain in hip and/or groin; ADL, function in daily living; Sport, function in sport and recreation; PA, participation in physical activities; QOL, hip and/or groin-related quality of life.

### Floor and ceiling effects

The frequencies of floor and ceiling effects are presented in Table 6. The prevalence of the minimal and maximum scores was lower than 15% [23].

**Table 6:** Frequency of the minimum and maximum scores on the HAGOS-NL (N = 117).

	Mean (SD)	Median	Floor effect	Ceiling effect
Symptoms	56.5 (25.1)	60.7	2 (1.7%)	5 (4.3%)
Pain	61.9 (26.0)	60.0	0 (0.0%)	9 (7.7%)
ADL	57.3 (28.4)	57.3	2 (1.7%)	7 (6.0%)
Sport*	41.6 (30.1)	41.6	6 (5.1%)	4 (3.4%)
PA	40.5 (28.2)	40.5	15 (12.8%)	9 (7.7%)
QOL	45.1 (24.4)	45.1	2 (1.7%)	3 (2.6%)

Floor and ceiling effects are reported as N (%).

* N = 116.

Abbreviations: Symptoms, symptoms in hip and/or groin; Pain, pain in hip and/or groin; ADL, function in daily living; Sport, function in sport and recreation; PA, participation in physical activities; QOL, hip and/or groin-related quality of life.

## Discussion

This study aimed to determine validity and reliability of the HAGOS-NL in a general population of middle-aged patients with hip complaints. Based on the results, it can be concluded that the HAGOS-NL is a valid and reliable questionnaire to assess pain, physical functioning and health-related quality of life in middle-aged patients with hip complaints. Factor analysis confirmed the structure of six subscales of the original HAGOS, which is in line with the studies that confirmed this structure for the Dutch, Danish, and Swedish versions of the HAGOS [10–12].

Construct validity was evaluated by defining 14 hypotheses about the magnitude of the relationship between the HAGOS-NL and the iHOT-12NL, HOOS and RAND-36. All hypotheses were confirmed, indicating good construct validity. As expected, correlations higher than 0.5 were found between the HAGOS-NL subscales and the total score of the iHOT-12NL. Although the correlation of the HAGOS-NL subscale *Functioning in physical activities* with the iHOT-12NL was higher than in the Swedish validation study [11] (0.65 and 0.37, respectively), in both studies the comparison yielded the lowest correlation. For the comparisons of the HAGOS-NL with the HOOS and RAND-36, all hypotheses were confirmed. This pattern was also confirmed in the other validation studies for the HAGOS [10–12].

The results also showed that physically active participants had higher scores on the HAGOS-NL than participants with lower activity levels. This is in line with previous research showing that physical activity is associated with improvements in various aspects of health-related quality of life in chronically diseased persons [27]. The HAGOS was originally developed for physically active patients, but as no floor effects were present it may also be suitable for less physically active patients. This suggestion is confirmed by sub-analysis in which active and inactive patients showed similar results.

Internal consistency of the HAGOS-NL proved to be good, with Cronbach’s alpha values ranging from 0.89 to 0.98. These values are comparable with the Dutch, Swedish and Danish validation studies of the HAGOS [10–12]. However, most subscales have Cronbach’s alpha values exceeding 0.95, which indicates that one or more items in those subscales might be redundant [19,28].

Test-retest reliability of the HAGOS-NL is considered good, with ICC values of 0.80 and higher. These values are comparable with ICC values found in studies on the validity of other language versions of the HAGOS [10–12].

The SEM values (6.6 to 12.3) and low MDC values at the group level (2.3 to 4.4) are comparable to SEM and MDC values found by Thorborg et al. (2011), Thomee et al. (2013) and Brans et al. (2016) [10–12]. Low MDC values at the group level indicate that the HAGOS-NL is sufficient for group comparisons, as only low values are needed to detect change. However, only values higher than the SEM can be distinguished from the measurement error, therefore to detect a statistically significant change in scores on the HAGOS-NL the difference should be higher than the SEM. To assess whether the group difference is also clinically important, the Minimal Important Change (MIC) should be determined [19]. Hence, for evaluative purposes, the MIC of each HAGOS subscale should be determined in future research.

MDC values at the individual level ranged from 18.3 to 34.1. In order to distinguish from a measurement error and to confirm a real change occurred, the difference between two measurements should be greater than the MDC_ind_ value. It is therefore questionable whether the HAGOS-NL is an appropriate tool for monitoring health-related quality of life and physical function in an individual patient over time. This is supported by the studies of Thomee et al. (2013), Thorborg et al. (2011), and Brans et al. (2016), who showed floor and/or ceiling effects for the HAGOS [10–12]. However, the current study found that no more than 15% of the participants achieved the lowest or highest possible score on one of the subscales of the HAGOS-NL, which supports recommending the HAGOS-NL to detect improvement and deterioration over time. Differences in findings of floor and ceiling effects may be explained by differences in patient populations. Future research should consider whether the HAGOS-NL is suitable for measuring changes in health status of a patient with hip complaints during a given time interval.

This study included a relatively high percentage of inactive patients (69%). Because the HAGOS was initially designed for an active patient group (Tegner score ≥4), floor effects may be expected, but were not found. Sub-analysis with physically active and inactive patients showed similar results, suggesting that the HAGOS-NL may also be suitable for inactive patients. However, more research is needed to draw definite conclusions.

In conclusion, the HAGOS-NL is a reliable and valid instrument for measuring pain, physical functioning and quality of life in middle-aged patients with hip complaints.

## Supporting information

S1 Dataset (SAV)
